# The Action of Certain Somnifacients on the Horse1Read before the Pennsylvania State Veterinary Medical Association, March 7, 1900.

**Published:** 1900-04

**Authors:** E. Stanton Muir

**Affiliations:** Philadelphia, Pa.


### THE JOURNAL

OF

### COMPARATIVE MEDICINE AND VETERINARY ARCHIVES.

VOL. XXI. *APRIL, 1900.* No. 4.

### THE ACTION OF CERTAIN SOMNIFACIENTS ON THE HORSE.[1]

BY E. STANTON MUIR, PH.G., V.M.D.,
PHILADELPHIA, PA.

THE action, on the human family, of those drugs termed somnifacients is sometimes accompanied by delirium, while in the majority of cases they have no other action than that of an hypnotic. On the horse and other solipeds they might be more correctly termed delirifacients, as their action is accompanied by a much more extended period of excitability; in fact, the somnolent action is often entirely disguised by the primary period of delirium, or the excitability produced by the drug is so great that its soporific action on the brain is entirely overcome. The comparative difference in the development between the brain and spinal cord in man is greater than that in the horse, and this has always seemed to me to be the reason of this marked difference in the action of those drugs whose force is directed primarily to the nervous system. We know that these drugs will act first on that portion of the nervous system which is best developed, and, for this reason, we can account for the pronounced difference in their action on the horse from that on man.

The physiological action of drugs on the horse is not given the study and attention that it merits. We give too many drugs to our patients (I refer to the horse), expecting certain results from our knowledge of their physiological action on man or from experiments on the dog, but not from a thorough knowledge gained from experiments on the horse. Again, we apply certain forms of medication to the horse and look for certain results, not because we have a rational reason for expecting such results, but because we know from former personal experience or from the clinical experience of

[1] Read before the Pennsylvania State Veterinary Medical Association, March 7, 1900.

others that such results are likely to follow. I am aware that this smacks of empirical therapeutics, but however much it may be to the discredit of the advanced veterinarian of to-day it is nevertheless true. We take advanced courses in surgery, practice, bacteriology, sanitary science, etc., all of which redound to the credit of the school adopting such advanced teaching, tend to further elevate our profession, and make more thorough, more intelligent, and more successful veterinarians, but this important part of a veterinarian's education—veterinary therapeutics—is neglected.

I cite these facts to bring as forcibly as possible to our notice the necessity for more experimental work in this branch.

Those drugs which produce sleep or whose chief physiological action is that of producing somnolence, are classed under various names: soporifics, hypnotics, narcotics, somnifacients, etc., but because the last name, somnifacients, translated means "sleep-producer," we have adopted this name for the drugs used in this line of experiments and have named this paper "The Physiological Action of Certain Somnifacients on the Horse." This paper details the physiological action of three drugs as shown in a series of experiments on fifteen horses.

The drugs used were P. & W.'s Sulphate of Morphia; P. D. & Co.'s normal liquid Indian Cannabis, and Schering's Crystals of Chloral. In some cases the drugs were administered subcutaneously, but in most cases intravenous injections were made, as we, of course, obtained quicker and more satisfactory results from the latter method of administering the drugs.

I wish here to speak a few words in favor of the administration of therapeutic agents by the intravenous and subcutaneous methods. The results are obtained much quicker, the drug can be administered more easily, and with average care the danger is almost *nil*. During this and another line of experiments there were a total of about eighty injections made, most of them intravenous, without the formation of a single abscess or any other untoward result. All the antiseptic precautions used were to have clean needles and sponge the skin with carbolic acid solution, 5 per cent., before making the injection. In fact, I do not think there is any more, if as much, danger in these forms of administering drugs as there is of the end of a drenching bottle breaking off and being swallowed by the animal when your remedial agent is administered by the mouth.

MORPHIA—EXPERIMENT No. 1. Bay gelding, fifteen or sixteen years old, fifteen and one-half hands, weighing 760 pounds. Temperature, 37.3° C. (99.2° F.); pulse soft and regular; respirations

6; heart, 35, regular, and impact good. Animal was poorly nourished. The first subcutaneous injection of 0.2 (3 grs.) of morphia sulphate was made at 11.17 A.M., and from that time until 2.59 P.M., three hours and forty-two minutes, he received in all 1.2 (18 grs.) of the salt in 0.2 doses. There was very little change in the animal until 1.07 P.M., a few minutes after the third dose of 0.2 (3 grs.) was given, at which time he became very restless and excitable. The pulse at this time was 50, full and soft, the heart irregular, and the respirations increased to 10. At 1.45 P.M. there was a partial loss of coördination; he was staggering, decidedly delirious, but not sleepy. The respirations became more and more labored. A profuse ptyalism was noted at 1.29 P.M., which continued with more or less intensity up to 4.20 P.M. From this hour until 8 P.M. the abnormal symptoms gradually subsided, except an excitable condition when approached. The following morning there were no signs to show that he had been subjected to the action of any medicinal agent. At 2.30 P.M., after 1 had been given, in five 0.2 doses, the pulse rose to 60; was full but soft; heart was rapid and very irregular, and at this time the excitability was most pronounced.

*The prominent symptoms* noticed during this experiment were great excitability, the least noise causing him to become extremely agitated, partial loss of coördination, ptyalism, labored respirations, and the action on the heart.

EXPERIMENT No. 2. Gray gelding, eight years old, fifteen and one-half hands high, weighing 800 pounds. Temperature, 37.7° C. (100° F.); pulse, heart, and respirations normal. General condition of the animal was poor. In this experiment the sulphate of morphine, in aqueous solution, was injected directly into the jugular vein. The first injection of 0.165 was made at 12.56 P.M. From this time until 2.48 P.M. (one hour and fifty-two minutes) I injected 1.09 (16.3 grs.) of the salt in doses not exceeding 0.2. At this hour (2.48) the pupils, which previously were normal or slightly contracted, became dilated; the pulse ran up to 84, and was almost imperceptible; he began to tremble all over the body, and became decidedly delirious. These symptoms gradually subsided until at 3.05 P.M. there was only a slight uneasiness noticed.

At 3.21 and at 3.51 I gave two more 0.2 doses, and at 4.05 P.M. he was in the same excitable condition noticed in Experiment No. 1; was standing with his head pushed up in the corner of the stall when not approached or annoyed; there was a profuse flow of saliva. At 4.20 he had received in all 1.49 (22 grs.) of the salt, at which time the temperature was 102.8° F., pulse full and very rapid, heart irregular. All symptoms except the excitability gradually subsided, and at 8 P.M. he was only slightly nervous. Next morning his condition was normal.

The *prominent symptoms* were excitability, trembling, labored respiration, rapid heart, and profuse ptyalism.

EXPERIMENT No. 3. Inasmuch as the doses given in Experiments Nos. 1 and 2 were small and frequently repeated, I decided to try the action of one large intravenous dose, and for this purpose I secured a gray gelding, eight years old, fifteen and one-half hands high, weighing 800 pounds. The temperature, respiration, and pulse were normal, the heart was strong and impact good. His condition was fair. At 10.55 A.M. injected 1 (15.4 grs.) into the jugular, and in two minutes he became very restless ; at 11.15 he was decidedly delirious, very easily excited, would quid his hay ; the pupils were widely dilated ; the pulse was full and rapid, the heart regular and 84. At 1 P.M., one hour and five minutes after the administration of the drug, the action had about passed off, and his condition was about normal.

The *prominent symptoms* noted here were not unlike those seen in Experiments Nos. 1 and 2, viz. : excitability, delirium, but no sleep, dilated pupils ; but in this case I noticed no flow of saliva, nor did the symptoms last so long.

EXPERIMENT No. 4. The last experiment with this salt was on a bay gelding, thirteen years old, fifteen and a quarter hands high, weighing 1050 pounds. Temperature 37.4° C., pulse 48, respirations 12, heart normal, impact good, and his general condition very good. In this case I injected 1 of sulphate of morphia, in aqueous solution, into the jugular at 12.04 P.M., and from that time until 2 he showed all those nervous symptoms which characterized the whole line of experiments, the marked excitability showing almost immediately after administering the drug, and increasing in intensity until 1 P.M., when it slowly subsided. A staggering gait was noticed five minutes after the injection, and the pupil was dilated. At 12.17 P.M. a tetanic condition existed for a short time, from 12.30 until 12.49, shown by a throwing back of the head and elevation of the tail. Ptyalism was profuse, starting five minutes after giving the injection, and continuing until 2.15 P.M. At 12.10 the pulse rose to 66, but was strong, and at 12.17 P.M. it jumped to 84. At 2 P.M. the temperature had risen to 38.1° C., the heart was 84 and strong, and the respirations were labored.

The *prominent symptoms* noticed were the characteristic excitable condition, staggering gait (loss of coördination), profuse ptyalism, dilated pupils.

The symptoms which showed in each of the experiments were great excitability, delirium, partial loss of coördination, and dilated pupils, and in all except No. 3 a profuse flow of saliva and a slight elevation of temperature.

CHLORAL, or as it was formerly known, hydrate of chloral, was the next drug experimented with. I used this on three horses. The solution used was chloralis, 150 grammes, dissolved in sufficient water to make 300 c.c.

EXPERIMENT No. 5 was on a bay gelding about fourteen years old, standing fifteen hands and three inches, and in fair condition. Temperature was 99.4° F., pulse normal, respiration 6, heart regular and fairly strong. General condition of this animal was fair. At 11.53 A.M. I injected an aqueous solution containing 15 (℥ss) of chloral into the vein of the neck. No change occurring up to 12.15 P.M., I again gave him the same quantity in the same manner. In five minutes after the second dose he was gaping constantly and swaying from side to side. At 1.25 P.M. the temperature had risen 1°, pulse was 60 and full, respirations increased from 6 to 12, but the heart was regular; he was standing quiet except some muscular tremors and was very sleepy. At 1.45 P.M. he received the third injection of 15 grammes, and from that time until 2.30 he was very sleepy and could barely stand, was constantly swaying from side to side, and his head was bobbing. At 3 P.M. he began to arouse, and all symptoms except drowsiness had passed.

*Prominent symptoms* were sleepiness, with a slight increase in temperature and respirations.

EXPERIMENT No. 6. Bay gelding, aged, sixteen and a quarter hands high. Temperature 99° F., pulse 36, respirations 36; heart slow, full, and regular. General condition poor. At 12 noon I injected 30 grammes of chloral into the jugular, and up to 12.28 P.M. noted no change in his condition. At that time I gave him the second 30, and in two minutes he fell down, I thought, to die, but it was only deep sleep, which continued until 12.52, when he unsuccessfully attempted to rise. During this time the respirations increased to 24, but were deep and regular, not jerky. The heart increased in frequency up to 72, and was labored. Up to 1.30 he had several times made unsuccessful attempts to rise, would drop off into a sleep, from which he was easily aroused. At this hour the temperature was the same, pulse was 36, respirations

30, deep and labored, and the heart regular. At 2.30 he arose with a little help, and the sleepy condition soon passed off, but the muscular weakness continued for several hours.

The *prominent symptoms* were sleep and muscular weakness, increase in frequency of the heart's action and respiration.

EXPERIMENT No. 7. Bay gelding, twelve years old, fifteen and one-half hands high, in good condition. Temperature 99.2° F., pulse 44, respirations normal; heart regular and strong. At 12 noon I administered 30 grammes chloral intravenously, which in four minutes caused the respirations to become deep, and all the symptoms recorded in the last experiment were noted. The heart became weak and irregular shortly after administering the drug. At 1.30 P.M. the temperature had risen to 101° F., the pulse to 60, respirations to 26, and the heart had become regular. The animal was standing quiet. At 1.45 he received 15 grammes of chloral, which caused him to sway from side to side and almost fall down in ten minutes. At 2.15 he was standing quiet, but would stagger when annoyed or on attempting to move. At 2.45 the symptoms were abating, and at 5 o'clock he was in about his normal condition and eating hay.

The *prominent symptoms* were like those of the other two experiments—sleep and muscular weakness, slight increase in temperature, respiration, and pulse.

The symptoms noticed in each of these experiments were sleep and apparent muscular weakness, and a slight increase in temperature. According to the best authorities, chloral kills by causing a loss of temperature, so when we remember the fact that we did not have a fall in temperature, even after we gave 60 grammes in the space of thirty minutes, we can feel sure that we were not near the toxic point of the drug, and that it is perfectly safe to give that amount in one hour's time intravenously in two doses.

(To be continued.)

